# Supplementary material for: Age-Specific Mortality Forecasting in Kazakhstan: Alternative Approaches to the Lee–Carter Model
Source: Int J Environ Res Public Health. 2025 Feb 26;22(3):346. doi: 10.3390/ijerph22030346 (PMC11941791; doi:10.3390/ijerph22030346)
Supplement: Supplementary file 1 [file ijerph-22-00346-s001.zip › ijerph-3419215-supplementary.pdf]

Table 1S. Kazakhstan mortality historical data (in 2020 and 2021 the actual data is highlighted in red, the smoothed data is in blue)

	[0]	[1-4]	[5-9]	[10-14]	[15-19]	[20-24]	[25-29]	[30-34]	[35-39]	[40-44]	[45-49]	[50-54]	[55-59]	[60-64]	[65-69]	[70-74]	[75-79]	[80-84]	[85+]
1991	27.93	1.90	0.65	0.57	1.15	1.77	2.14	2.64	3.33	4.65	6.78	10.75	15.52	22.93	31.56	46.63	68.86	108.00	180.55
1992	25.42	1.81	0.60	0.53	1.18	1.83	2.28	2.65	3.45	4.93	7.29	10.89	15.30	23.54	31.95	49.04	63.72	110.10	191.12
1993	25.65	2.06	0.61	0.57	1.29	2.18	2.62	3.21	4.07	6.01	8.29	12.87	17.73	26.40	36.44	54.87	65.21	119.48	198.75
1994	25.56	2.00	0.58	0.54	1.25	2.11	2.76	3.17	4.22	6.04	8.64	13.18	18.24	28.25	37.94	58.43	67.48	134.12	247.78
1995	27.19	2.30	0.59	0.55	1.23	2.39	3.03	3.66	4.98	7.18	10.37	15.39	21.05	30.34	40.97	55.19	82.57	122.44	207.50
1996	25.79	1.99	0.53	0.56	1.22	2.48	3.22	4.01	5.48	7.56	10.94	15.65	21.89	31.18	42.67	55.47	83.24	123.61	207.66
1997	24.81	1.92	0.54	0.49	1.30	2.40	3.14	4.00	5.38	7.30	10.40	14.81	21.36	30.14	42.70	55.87	81.43	120.38	192.81
1998	22.10	1.88	0.55	0.54	1.24	2.48	3.34	4.04	5.21	7.21	10.29	14.47	21.19	30.49	42.95	57.93	81.42	123.65	191.16
1999	20.76	1.55	0.49	0.49	1.18	2.20	3.07	3.68	4.70	6.42	9.17	13.15	19.84	28.98	41.91	57.31	80.20	119.11	192.72
2000	19.76	1.55	0.49	0.47	1.22	2.28	3.25	3.98	4.91	6.83	9.62	13.91	20.27	29.62	42.68	59.21	80.20	120.76	174.86
2001	19.54	1.36	0.47	0.49	1.13	2.37	3.04	3.74	4.87	6.96	10.03	14.86	19.03	27.31	41.95	54.47	82.20	120.67	218.73
2002	17.16	1.27	0.46	0.48	1.06	2.12	3.00	3.71	4.83	6.89	9.79	14.21	19.61	28.42	38.80	57.50	79.53	122.97	231.68
2003	16.02	1.20	0.46	0.46	1.08	2.09	3.00	3.88	4.84	7.08	10.14	14.41	20.21	29.28	40.67	59.64	83.55	124.75	241.65
2004	14.83	1.04	0.43	0.45	1.08	2.23	3.36	4.25	5.16	7.24	10.13	14.23	19.56	27.91	38.38	56.47	78.85	114.64	227.65
2005	15.45	1.12	0.47	0.46	1.08	2.29	3.50	4.55	5.46	7.36	9.99	14.42	19.66	28.30	39.02	55.06	80.89	115.22	236.24
2006	14.48	1.16	0.47	0.49	1.07	2.21	3.58	4.71	5.53	7.40	10.00	13.96	19.28	26.35	37.84	53.44	80.81	115.10	223.92
2007	15.07	1.07	0.46	0.53	1.15	2.12	3.54	4.75	5.57	7.26	9.51	13.53	18.31	26.00	36.77	51.92	81.06	116.97	229.28
2008	21.79	0.94	0.43	0.41	1.06	1.92	3.01	4.15	4.83	6.17	8.43	11.71	16.74	23.51	35.73	49.96	78.39	117.39	223.52
2009	18.54	0.91	0.40	0.35	0.89	1.63	2.43	3.47	4.27	5.54	7.52	10.66	15.45	22.32	33.46	47.95	73.27	109.54	200.66
2010	16.96	0.88	0.39	0.40	0.86	1.45	2.26	3.35	4.34	5.44	7.58	11.06	16.13	23.24	32.98	49.69	76.13	122.28	203.45
2011	15.11	0.86	0.33	0.38	0.77	1.45	1.98	3.16	4.14	5.20	7.20	10.64	15.15	22.74	32.26	48.50	74.53	120.70	206.35
2012	13.75	0.75	0.36	0.31	0.77	1.29	1.97	2.99	4.08	5.14	6.78	10.01	14.79	22.37	30.50	47.16	71.51	122.56	204.99
2013	11.56	0.76	0.30	0.33	0.74	1.24	1.78	2.76	3.86	4.84	6.29	9.16	13.59	20.52	28.03	44.52	67.68	113.40	200.58
2014	9.99	0.69	0.30	0.31	0.72	1.02	1.55	2.45	3.47	4.51	5.91	8.55	12.89	18.98	28.01	42.72	66.18	107.67	196.96
2015	9.43	0.69	0.31	0.34	0.67	1.02	1.40	2.26	3.32	4.33	5.78	8.15	12.31	18.60	27.40	41.26	64.55	102.83	197.17
2016	8.79	0.57	0.28	0.30	0.62	0.89	1.28	1.94	3.09	4.23	5.45	7.94	11.78	18.25	27.94	39.75	63.89	102.70	198.93
2017	7.92	0.57	0.28	0.29	0.61	0.92	1.17	1.78	2.85	3.94	5.33	7.37	11.12	17.43	26.61	38.08	62.87	97.88	196.42
2018	8.14	0.53	0.27	0.29	0.61	0.88	1.14	1.67	2.68	3.81	5.32	7.42	11.29	17.26	26.51	36.72	61.18	96.91	195.09
2019	8.47	0.60	0.28	0.29	0.59	0.91	1.08	1.69	2.69	3.99	5.48	7.29	10.91	17.30	26.00	37.77	60.33	97.63	191.30
**2020**	7,77	0,47	0,22	0,25	0,53	0,83	1,02	1,71	2,72	4,41	6,23	8,86	13,19	21,26	32,12	49,25	74,05	120,24	218,00
**2021**	8,44	0,48	0,24	0,30	0,61	0,90	1,17	1,72	2,77	4,55	6,52	9,23	13,82	23,03	37,11	58,84	84,45	137,59	237,07
2020	8.21	0.57	0.27	0.28	0.58	0.85	1.04	1.63	2.57	3.94	5.38	7.26	10.60	16.78	25.06	36.87	3.94	5.38	7.26
2021	7.94	0.54	0.26	0.28	0.58	0.80	1.01	1.58	2.45	3.90	5.27	7.23	10.28	16.26	24.11	35.96	3.90	5.27	7.23
2022	7.68	0.51	0.25	0.27	0.57	0.74	0.97	1.52	2.33	3.85	5.17	7.20	9.97	15.74	23.17	35.06	52.23	90.22	149.93
2023	7.63	0.54	0.26	0.27	0.57	0.77	0.98	1.43	2.15	3.53	5.25	7.10	9.88	15.26	22.35	33.70	49.16	82.38	129.53

Table 2S. Kazakhstan mortality forecasting data

	[0]	[1-4]	[5-9]	[10-14]	[15-19]	[20-24]	[25-29]	[30-34]	[35-39]	[40-44]	[45-49]	[50-54]	[55-59]	[60-64]	[65-69]	[70-74]	[75-79]	[80-84]	[85+]
2024	7	0.52	0.23	0.24	0.6	0.78	0.95	1.42	2.17	3.34	4.68	6.55	9.54	15.06	22.53	33.84	51.55	87.51	154.23
2025	6.36	0.5	0.21	0.23	0.58	0.74	0.9	1.36	2.1	3.24	4.53	6.33	9.25	14.66	22	33.12	50.71	86.42	152.92
2026	5.73	0.48	0.19	0.22	0.56	0.71	0.85	1.3	2.03	3.14	4.39	6.12	8.96	14.26	21.48	32.41	49.88	85.34	151.61
2027	5.09	0.47	0.18	0.21	0.54	0.67	0.8	1.24	1.96	3.04	4.25	5.91	8.68	13.88	20.97	31.72	49.07	84.28	150.32
2028	4.46	0.45	0.16	0.2	0.52	0.64	0.76	1.19	1.9	2.95	4.12	5.71	8.41	13.5	20.47	31.04	48.27	83.23	149.04
2029	3.82	0.43	0.15	0.19	0.5	0.61	0.72	1.14	1.83	2.86	3.99	5.52	8.15	13.14	19.99	30.38	47.49	82.2	147.77
2030	3.19	0.42	0.14	0.18	0.49	0.58	0.68	1.09	1.77	2.77	3.86	5.34	7.9	12.78	19.52	29.73	46.71	81.17	146.51
2031	2.56	0.4	0.13	0.17	0.47	0.55	0.64	1.04	1.71	2.69	3.74	5.16	7.65	12.44	19.05	29.09	45.95	80.16	145.27
2032	1.92	0.38	0.11	0.16	0.45	0.52	0.61	0.99	1.66	2.61	3.62	4.98	7.42	12.1	18.6	28.47	45.2	79.17	144.03
2033	1.29	0.37	0.1	0.15	0.44	0.49	0.57	0.95	1.6	2.53	3.5	4.82	7.19	11.77	18.16	27.86	44.47	78.18	142.8

Section 1S Stationarity testing
1. Automatic stationarity test
auto.arima() first performs a stationarity test using:
• KPSS test (default)
KPSS test checks whether the series is stationary (null hypothesis H_0: the series is stationary).
If p-value < 0.05 → the series is non-stationary.
If p-value ≥ 0.05 → the series is stationary.
If the time series is not stationary, the first differentiation is performed d =1.
If the series is still non-stationary after d = 1, the function increases d and tries d = 2. If the series remains non-stationary at d = 2
There are two possible cases:
1. If the series has become stationary after d = 2, auto.arima() uses this order (ARIMA(p, 2, q)).
2. If even after d = 2 the series is non-stationary, then:
• The function may stop with an error (Error: No ARIMA model able to be fitted).
• Or it will choose the ARIMA model with d = 2, but warns that the model may be unstable.
Our results

Before differencing

Age	KPSS p-value	
**0**	0.01	non-stationary
**1**	0.012909532	non-stationary
**2**	0.01	non-stationary
**3**	0.01	non-stationary
**4**	0.012558122	non-stationary
**5**	0.019364202	non-stationary
**6**	0.032333491	non-stationary
**7**	0.053361763	non-stationary
**8**	0.026089447	non-stationary
**9**	0.021581761	non-stationary
**10**	0.032283686	non-stationary
**11**	0.022939072	non-stationary
**12**	0.022411642	non-stationary
**13**	0.020407784	non-stationary
**14**	0.023121124	non-stationary
**15**	0.021509607	non-stationary
**16**	0.02886279	non-stationary
**17**	0.01422059	non-stationary
**18**	0.064335087	non-stationary

After 1st differencing

Age	KPSS p-value	
**0**	0.1	stationary
**2**	0.1	stationary
**3**	0.1	stationary
**4**	0.1	stationary
**5**	0.073519978	stationary
**6**	0.088671892	stationary
**7**	0.076582585	stationary
**8**	0.036891862	non-stationary
**9**	0.077191175	stationary
**10**	0.09783471	stationary

**11**	0.1	stationary
**12**	0.084485437	stationary
**13**	0.078970602	stationary
**14**	0.054362755	stationary
**15**	0.023514683	non-stationary
**16**	0.045079944	non-stationary
**17**	0.1	stationary
**18**	0.1	stationary

After 2nd differencing

Age	KPSS p-value	
**8**	0.1	stationary
**15**	0.1	stationary
**16**	0.1	stationary
